# Time-resolved transcriptomics of haemocyte discrimination between challenges by nematodes and inert material in the oriental armyworm *Mythimna separata*

**DOI:** 10.3389/fimmu.2026.1810925

**Published:** 2026-04-29

**Authors:** Masaya Ono, Xiaolan Li, Yasunobu Maeda, Rei Nishimura, Jion Tsuruta, Akemi Yoshida, Kazuki Sato, Taisei Kikuchi

**Affiliations:** 1 Laboratory of Parasite Systems Biology, Department of Integrated Biosciences, Graduate School of Frontier Sciences, The University of Tokyo, Kashiwa, Chiba, Japan; 2 Department of infectious disease, Faculty of Medicine, The University of Miyazaki, Miyazaki, Japan; 3 Genomics and Bioenvironmental Science, Frontier Science Research Center, University of Miyazaki, Miyazaki, Japan

**Keywords:** encapsulation, haemocyte, immunity, insect, nematode, RNA-seq

## Abstract

**Introduction:**

Insects defend against large metazoan parasites such as nematodes through haemocyte-mediated encapsulation, but how haemocytes respond to living parasites remains poorly understood. This study examined the cellular and transcriptional responses of haemocytes to living nematodes in the lepidopteran insect *Mythimna separata*.

**Methods:**

Encapsulation assays were combined with time-course transcriptomic analyses to compare haemocyte responses to inert polystyrene beads and the free-living nematode *Caenorhabditis elegans*. Haemocytes were collected following injection treatments, and RNA sequencing was used to characterise temporal changes in gene expression.

**Results:**

Inert polystyrene beads were rapidly encapsulated, whereas *C. elegans* induced a slower, progressive encapsulation response. Time-resolved RNA sequencing showed that injection alone established a structured physiological baseline, while nematode exposure consistently drove haemocytes into distinct transcriptional states not observed with inert beads. Nematode-associated responses were enriched for regulatory, immune, and membrane-related processes, with biased and temporally stabilised engagement of the Toll signalling pathway. Importantly, Toll pathway activation also occurred in response to axenically prepared nematodes, demonstrating that living nematodes alone are sufficient to influence canonical innate immune signalling.

**Discussion:**

These findings show that haemocytes distinguish living nematodes from inert particles not merely through their physical presence, but by mounting sustained and coordinated transcriptional responses. This study provides a temporal and mechanistic framework for understanding how insect immune systems recognise and respond to large metazoan parasites.

## 1 Introduction

In natural environments, insects are constantly exposed to a wide array of pathogens, ranging from microorganisms such as bacteria and fungi to large metazoan parasites including nematodes and parasitoid wasps. Among these, insect-parasitic nematodes, particularly members of the family *Mermithidae* and the genera *Steinernema* and *Heterorhabditis*, are one of the most severe threats to insect survival as infection often results in extensive tissue damage and host mortality (1–7). To counter nematode invasion, insects rely on a robust cellular immune response known as encapsulation, which is mediated by haemocytes (8–11). Five major types of haemocytes are generally recognised: granulocytes, plasmatocytes, oenocytoids, spherulocytes, and prohaemocytes (12). Granulocytes and plasmatocytes constitute approximately 70–80% of the total haemocyte population and play central roles in encapsulation (13–15). Encapsulation often occurs together with melanisation (16–18). During melanisation, activation of the phenoloxidase cascade leads to the production of melanin, which is deposited around the nematodes and contributes to its inactivation (17, 19–22). Oenocytoids are known to play a key role in the production of phenoloxidase during this process (12). Upon entry of nematodes into the haemocoel through natural openings such as the mouth, anus, or spiracles or directly through the cuticle, granulocytes and plasmatocytes adhere to the parasite surface and assemble into multilayered cellular aggregates that surround, immobilize, and ultimately neutralise the invading nematode, thereby executing the encapsulation response (10, 11, 13, 16, 23, 24).

Most of our current understanding of insect encapsulation mechanisms has been derived from studies using artificial targets, such as chromatographic or polystyrene beads (25–30), as well as parasitoid wasp eggs (31, 32). These systems have been instrumental in identifying key molecular players involved in haemocyte adhesion and aggregation. In several lepidopteran species, including *Manduca sexta*, *Helicoverpa armigera*, and *Mythimna separata*, C-type lectins have been shown to function as pattern recognition receptors that bind carbohydrate moieties on foreign surfaces and promote haemocyte attachment, thereby facilitating encapsulation (17, 18, 29, 30, 33–36). Similarly, integrins and tetraspanins expressed on haemocyte surfaces have been implicated in mediating adhesion to beads and parasitoid-derived targets (27, 28, 37–40).

However, artificial particles differ fundamentally from parasitic nematodes in size, surface composition, and biological activity. Unlike inert beads, nematodes are large, living organisms that actively interact with host tissues and may present distinct molecular cues to the immune system. Consequently, it remains unclear whether haemocytes recognise nematodes through the same molecular pathways defined in bead-based systems, or whether nematode encapsulation relies on additional or distinct recognition and response mechanisms. Despite the ecological and pathological significance of nematode infections, the genetic and molecular basis of haemocyte-mediated nematode encapsulation has received comparatively little attention, leaving a major gap in our understanding of how insects tailor cellular immune responses to complex metazoan parasites.

In contrast to the limited understanding of nematode encapsulation, recognition mechanisms underlying insect immune responses to microorganisms such as bacteria and fungi have been extensively characterised (41–43). In these systems, haemocytes and humoral immune pathways rely on well-defined pattern recognition receptors, including peptidoglycan recognition proteins, β-glucan recognition proteins, and C-type lectins, to detect conserved microbial surface components and activate downstream signalling pathways such as the Toll and Imd pathways. These studies have established a paradigm in which relatively small pathogens are recognised through conserved molecular patterns, leading to rapid and stereotyped immune responses. Whether this paradigm extends to the recognition of large, multicellular parasites such as nematodes remains unclear.

In this study, we set out to elucidate the molecular and temporal basis of haemocyte-mediated encapsulation of nematodes. Using the lepidopteran insect *M. separata* as a model, we examined encapsulation responses to the free-living nematode *Caenorhabditis elegans*, alongside inert beads, as experimentally tractable targets that differ fundamentally in biological complexity. Despite not being an obligate insect parasite, *C. elegans* provides a genetically tractable, living multicellular target with defined size and surface properties, enabling controlled analysis of haemocyte responses to a large metazoan organism. In contrast, studies using entomopathogenic nematodes (EPNs) have shown that exposure to the nematodes themselves, as well as to their excreted–secreted (ES) products or extracellular vesicles (EVs), can modulate host immune gene expression in Drosophila, including antimicrobial peptides such as Attacin and Diptericin and related signalling pathways, in a system-dependent manner (44–46). In such settings, it is difficult to disentangle transcriptional changes elicited by the nematodes themselves from those induced by nematode-derived immunomodulatory molecules. In addition, the limited availability of genetic tools in these nematodes constrains mechanistic dissection of host responses. For these reasons, axenically prepared *C. elegans* provides a useful reductionist system for examining haemocyte responses to a living multicellular target while minimizing these additional confounding factors.

Using this system, we compared encapsulation responses to live nematodes and inert polystyrene beads, revealing marked differences in the kinetics of haemocyte recruitment and capsule formation. To determine whether these differences reflect delayed activation of shared pathways or engagement of distinct recognition mechanisms, we integrated time-course transcriptomic profiling with clustering and differential expression analyses to identify stimulus-specific transcriptional programs associated with early recognition and later execution phases of encapsulation.

## 2 Materials and methods

### 2.1 Insects and nematodes

Armyworms, *M. separata* (strain Saga) larvae were reared on an artificial diet (Insecta LFM, Nosan Corporation, Yokohama, Japan) at 25 °C in a plastic chamber, and the adults were maintained on a honey solution at 25 °C in cardboard cages placed inside an incubator. The last instar (6th) larvae were used in the experiments. *C. elegans* N2 obtained from the Caenorhabditis Genetics Center (University of Minnesota, St. Paul, MN, USA) were maintained on nematode growth medium (NGM) plates (1.7% agar) seeded with *Escherichia coli* strain OP50 following standard procedures (47) at 20 °C. To obtain axenic, synchronised L1 larvae, gravid adult hermaphrodites were collected from NGM plates and subjected to alkaline hypochlorite treatment (1% sodium hypochlorite; 0.2M NaOH) to lyse adult bodies and isolate embryos. The embryos were incubated overnight in M9 buffer (3 g of KH_2_PO_4_, 6 g of Na_2_HPO_4_, 5 g of NaCl, 1 mL of 1 M MgSO_4_, H_2_O to 1 L) at 20 °C in the absence of food to allow hatching and entry into L1 developmental arrest. Hatched L1 larvae were then processed using modified Baermann method (10) to obtain active L1 larvae and washed five to six times with M9 buffer.

### 2.2 Encapsulation assay

Fifty microliters of nematode suspension containing 200–300 sterile, developmentally arrested L1 larvae of *C. elegan*s in 50 μm of M9 buffer was injected into individual insect larvae using a 1 mL tuberculin syringe fitted with a 26 G × 1/2′′ needle (SS-01T2613S, Terumo Corporation, Tokyo, Japan). Insects were dissected under a microscope at 2, 3, 4, 5, 7 and 14 h post injection. To recover the injected nematodes, the insect carcasses were carefully washed with M9 buffer, and recovered nematodes were counted using a Syracuse clock dish (Fujiwara Scientific Co., Osaka, Japan) and scored as encapsulated or unencapsulated. At each time point, three insects were analysed and the experiment was independently repeated three times. For each time point, encapsulation rates were first calculated for individual insects. Within each independently repeated experiment, the values from three insects were averaged to generate one experiment-level mean. These means from three independent experiments were treated as biological replicates (n = 3). As an inert control for encapsulation, 500–600 polystyrene beads (90 μm in diameter), Polysciences, PA, USA) in 50 μm of M9 buffer were injected into individual insect larvae using the same procedure. Recovered nematodes and beads were also examined under a stereomicroscope, and representative images were captured to visually confirm hemocyte-mediated encapsulation. The encapsulation assay was used primarily to describe the temporal progression of haemocyte coverage in each treatment.

### 2.3 Assessment of bacterial contamination in nematode suspensions

To evaluate potential bacterial contamination in nematode suspensions after bleach treatment, bacterial culture assays and detection of 16S rRNA genes were performed using the supernatant of nematode suspensions. Briefly, 500 μL of the supernatant from nematode suspensions 24 h after bleach treatment was plated onto LB agar plates (Sigma-Aldrich, St. Louis, MO, USA) and incubated at 37 °C. As controls, M9 buffer or the supernatant of nematode suspensions immediately collected from NGM plates without bleach treatment were processed in parallel. For molecular detection, genomic DNA in the supernatants was analysed using universal 16S rRNA gene primers by conventional PCR and quantitative PCR (qPCR). Conventional PCR was performed using KAPA HiFi HotStart ReadyMix (Roche, Basel, Switzerland). Amplification products were confirmed using an Agilent TapeStation system.

### 2.4 Assessment of haemocyte composition

At 2 and 10 h post-treatment (non-injected, buffer-, bead-, and nematode-injected), haemolymph was collected by severing a proleg and haemocytes were recovered. Collected haemocytes were observed using a OneCell Counter (OneCell Co., Ltd., Shiga, Japan) and classified into granulocytes, plasmatocytes, and other cell types based on morphological characteristics. For each sample, the number of each haemocyte type was counted, and their relative proportions were calculated against the total haemocyte count. Five insects were analysed individually per condition and time point in each experiment, and the experiment was independently repeated three times. Each experimental repeat was treated as a biological replicate. Statistical analyses were performed using R. Total haemocyte number was analysed separately at 2 h and 10 h using negative binomial generalized linear models with Condition as the explanatory variable. Haemocyte composition (Granulocyte, Plasmatocyte, and Other) was analysed separately at each time point by Dirichlet regression using Condition as the explanatory variable. For compositional analysis, overall condition effects were evaluated by likelihood ratio tests comparing the full and intercept-only models. All tests were two-sided, and significance was defined as P < 0.05; *post hoc* contrasts were corrected for multiple testing.

### 2.5 RNA extraction

At 1, 2, 5, and 10 h after injection of 200–300 nematodes, 500–600 polystyrene beads, or 50 μL of M9 buffer, haemolymph containing haemocytes was collected by severing a proleg and released directly into a 1.5 mL tube containing 200 μL of TRIzol reagent (Sigma-Aldrich, St. Louis, MO, USA). One biological replicate consisted of pooled haemolymph from two insects. Three independent biological replicates were prepared for each condition and time point, and no technical replicates were included. Haemolymph was also collected from non-injected insects. In total, 39 samples were analysed: non-injected controls (n = 3) and buffer-, bead-, and nematode-injected samples (4 time points × 3 biological replicates per condition; n = 12 per condition). Haemocytes suspended in TRIzol reagent were manually homogenized using the disposable pestle provided with BioMasher II (Nippi), with intermittent snap freezing to ensure complete cell disruption. TRIzol reagent was added to a final volume of 500 μm, followed by the addition of 100 μm of chloroform. Samples were mixed and centrifuged at 12, 000 × g for 15 min at 4 °C. The aqueous phase was transferred to a new 1.5 mL tube, mixed with 250 μm of 2-propanol, and centrifuged at 12, 000 × g for 15 min at 4 °C. The RNA pellet was washed with 75% ethanol, air-dried for 1–2 min and resuspended in 20 μm of RNase-free water (Invitrogen, Carlsbad, CA, USA) and stored at -80 °C until use. RNA concentration was measured using a Qubit fluorometer (Thermo Fisher Scientific, Waltham, MA, USA), and RNA integrity was evaluated using an Agilent TapeStation (Agilent Technologies, Santa Clara, CA, USA). All samples exhibited RNA integrity number (RIN) values of ≥ 7 and were used for library preparation (**Supplementary Table 1**).

### 2.6 RNA-seq library preparation and sequencing

All RNA samples were submitted to Veritas Corporation (Hangzhou, China) for library preparation and sequencing. Stranded mRNA libraries were prepared using the Illumina Stranded mRNA Prep kit, and sequenced on an Illumina NovaSeq platform to generate paired-end 150 bp reads.

### 2.7 RNA-seq data processing and gene count generation

Raw RNA-seq read quality was assessed using FastQC (v0.12.1). No samples were excluded based on quality control criteria. Reads were aligned to the reference genome (GCA_029852925.1, NCBI) using HISAT2 (v2.2.1) (48). The resulting SAM files were converted to BAM format, sorted, and indexed using SAMtools. Gene-level read counts were calculated using HTSeq-count (v1.15) (49), and count matrices were constructed. Gene Ontology terms, KEGG pathways, and Pfam domain annotations were assigned using eggNOG-mapper (v2.1.9) (50).

### 2.8 Principal component analysis

Read count data were converted to transcripts per million (TPM) followed by log_2_ transformation with the addition of a pseudocount of 1. PCA was performed on the log_2_-transformed TPM expression matrix using the prcomp function in R (v4.3.1), with genes treated as variables and samples as observations. Samples were visualised using the first and second principal components, and the percentage of variance explained by each component was indicated on the axes.

### 2.9 Time-course clustering analysis

Time-course RNA-seq analysis was performed using the Bioconductor package moanin (48). Gene-level read count data together with sample metadata (experimental group, time point, and biological replicate) were used as input. To reduce noise and enrich for dynamically regulated genes, temporal variability was quantified for each gene using the median absolute deviation (MAD), and only genes within the top 80% of MAD values were retained for downstream analysis.

Temporal expression trajectories were modelled using spline regression with two degrees of freedom to capture non-linear transcriptional changes across time for each experimental condition. Differential time-course expression between conditions was evaluated using the DE_timecourse function, which tests for differences between spline-fitted expression trajectories across the full time course. This approach enables detection of genes exhibiting condition-dependent transcriptional dynamics rather than timepoint-specific differences alone.

Pairwise contrasts were performed between experimental groups (buffer vs bead, buffer vs nematode, and bead vs nematode) to identify genes showing distinct transcriptional responses across the entire temporal profile. To summarise statistical evidence across comparisons, p-values derived from individual contrasts were combined for each gene using Fisher’s method. Genes were considered dynamically regulated if they showed a combined p-value < 0.01 and a maximum absolute log2 fold change greater than 2 in at least one comparison. To ensure that biologically meaningful transcriptional changes were not overlooked by overly stringent statistical filtering, gene selection criteria were designed to balance statistical significance with biological relevance. Log2 fold changes were estimated using the timecourse method implemented in estimate_log_fold_change.

Genes with significant temporal regulation were grouped according to similarity in their expression trajectories using spline-based k-means clustering. The number of clusters was determined by evaluating clustering stability and structure across a range of K values (K = 2–7). Cluster stability was assessed using a resampling-based consensus approach, while clustering structure was evaluated using the elbow method and silhouette analysis based on distance metrics derived from the expression data. The final number of clusters was selected based on the combined outcomes of these criteria, together with consideration of biological interpretability.

### 2.10 Differential expression analysis

DE analysis was performed using edgeR (v3.42) in R (51). Prior to differential expression analysis, lowly expressed genes were filtered independently for each comparison by retaining genes with CPM > 1 in at least 2 samples. Of 17, 542 genes in the count matrix, 8, 205–8, 522 genes were retained depending on the comparison. Gene-level count data were analysed at each time point to quantify condition-specific transcriptional differences. Pairwise comparisons included non-injected controls versus buffer-injected samples (1, 2, 5, and 10 h), and bead- or nematode-injected samples versus time-matched buffer controls (1, 2, 5, and 10 h). Genes were considered significant if they met both criteria of FDR ≤ 0.05 and |log2 fold change| ≥ 1.0. Volcano plots were generated for each contrast by plotting log2 fold change against −log10(P-value). Significant genes were classified as upregulated or downregulated based on the sign of the log2 fold change and were highlighted accordingly. Overlaps between the bead and nematode conditions, including shared and condition-specific genes, were visualised using custom R scripts. Circle sizes were scaled proportionally to gene set size, and overlap areas represent the number of shared genes.

### 2.11 Gene ontology enrichment analysis

Gene Ontology (GO) enrichment results were summarised to reduce redundancy and improve biological interpretability using a semantic similarity–based clustering approach. GO enrichment analysis was initially performed using GOseq (52), which corrects for gene length bias in RNA-seq data. Enriched GO terms were filtered using a predefined significance threshold (p value <0.001). Semantic similarity among enriched GO terms was calculated using the Wang method implemented in the GOSemSim framework (53). This method evaluates functional similarity based on the hierarchical structure of the Gene Ontology directed acyclic graph (DAG). Pairwise semantic similarity scores were used to cluster GO terms into functional groups using the rrvgo framework. Within each semantic cluster, a representative GO term was selected based on statistical significance, defined as the highest −log10(p-value) among cluster members. This strategy prioritises biologically specific GO terms while preserving statistical robustness. Similarity thresholds 0.9 were applied to define clustering stringency, where higher threshold values resulted in stronger merging of semantically related GO terms. All analyses were performed using custom R scripts integrating GOSemSim and rrvgo algorithms (53, 54).

### 2.12 Heatmap analysis

Heatmaps were generated from gene-level read count data after converting counts to transcripts per million (TPM) to account for library size and gene length. For each gene and sample, TPM was calculated by normalising raw counts by gene length (in kilobases) and scaling by the total number of length-normalised reads per sample. The resulting TPM matrix was log2-transformed after adding a pseudocount of 1 (log2[TPM + 1]). For visualisation, values were then standardized per gene by row-wise Z-score scaling. The heatmap was drawn using the ComplexHeatmap package (v2.18) in R (53). Samples were ordered as Non-inject, Buffer (1, 2, 5, 10 h), Bead (1, 2, 5, 10 h), and Nematode (1, 2, 5, 10 h). Rows were split by the pre-defined cluster assignment, and both row and column clustering were disabled.

### 2.13 KEGG pathway analysis

KEGG pathway analysis was performed using KEGG Orthology (KO) annotations assigned by eggNOG-mapper. KO annotations were integrated with differential expression results obtained from edgeR. When a gene was associated with multiple KO terms, each KO was treated independently. For pathway visualisation, log2 fold change values were summarised at the KO level by averaging log2 fold changes when multiple genes mapped to the same KO. KO-level log2 fold changes were mapped onto KEGG pathway diagrams using pathview package (v1.40) in R (54). The Toll and Imd signalling pathway (ko04624) was visualised, and the color scale was fixed to a log2 fold change range of −2 to +2. Differential expression values were calculated by comparing nematode-injected samples with buffer-injected controls at the same time point, thereby isolating nematode-associated transcriptional responses from injection-induced effects.

## 3 Results

### 3.1 Temporal dynamics of nematode and bead encapsulation

**Figure 1:**
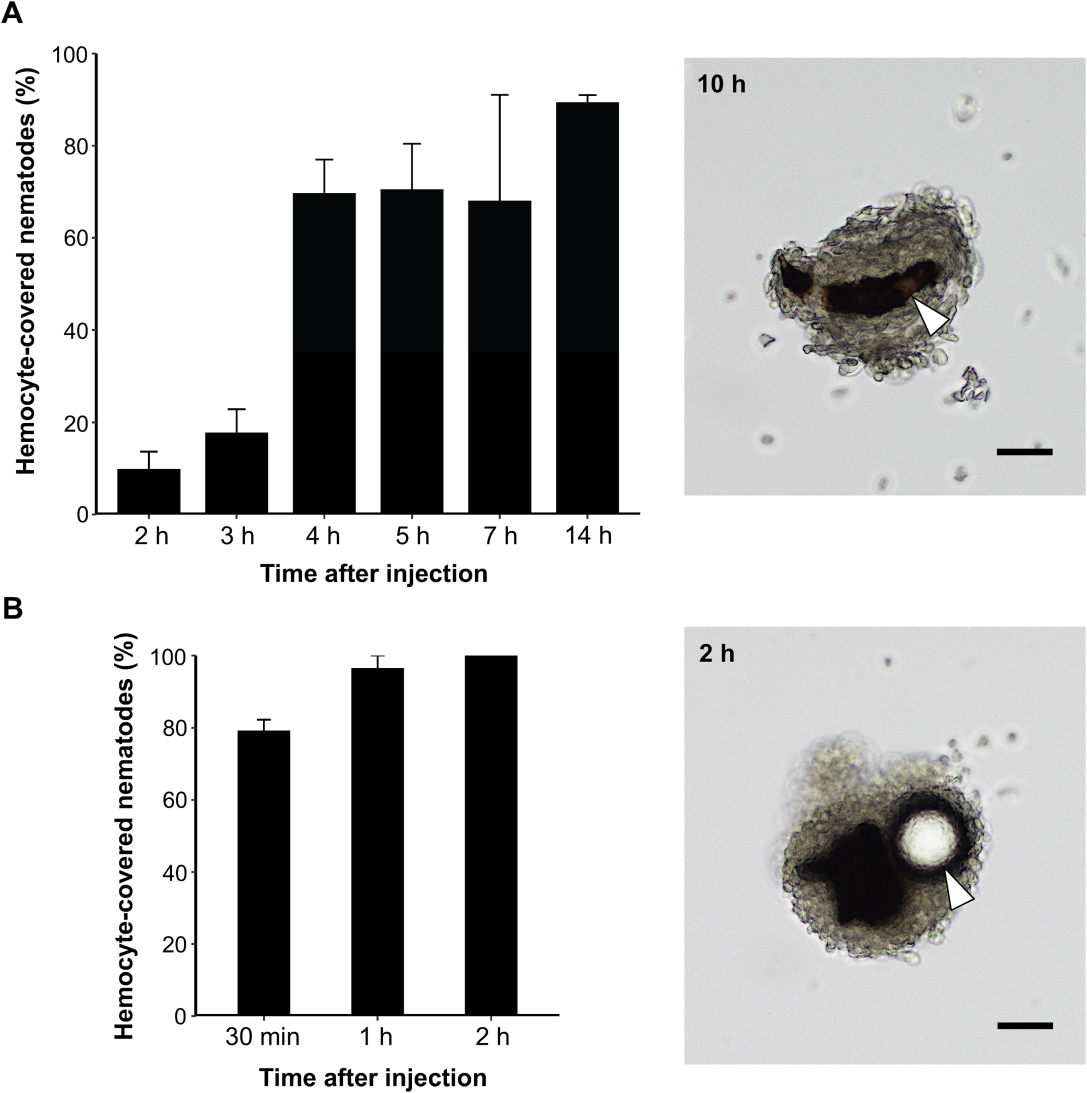
Haemocyte encapsulation kinetics following injection of nematodes and inert beads. **(A)** Percentage of nematodes covered by haemocytes at 2, 3, 4, 5, 7, and 14 h post-injection in *Mythimna separata*. **(B)** Percentage of beads covered by haemocytes at 30 min, 1 h, and 2 h post-injection. Representative stereomicroscopic images of haemocyte-mediated encapsulation are shown to the right of each percentage plot (2 h for beads and 10 h for nematodes). Nematodes and beads are indicated by arrowheads. Melanisation is visible as dark pigmentation surrounding the foreign material. Scale bar = 50 μm.

To characterise the dynamics of haemocyte-mediated encapsulation, we first quantified the temporal progression of the cellular immune response following injection of *C. elegans* into the insect haemocoel (**Figure 1A**). At early time points, haemocyte association with nematodes was limited, with only ~10% and ~20% of nematodes being haemocyte-covered at 2 h and 3 h post-injection, respectively. A marked increase in haemocyte association was observed from 4 h, with approximately 65–70% of nematodes encapsulated by haemocytes at 4–7 h. By 14 h post injection, nearly 90% of nematodes were encapsulated, indicating encapsulation of nematodes is a gradually established cellular immune response that requires several hours to reach full efficiency. Those nematodes were encapsulated with accompanying melanisation, as confirmed by stereomicroscopic imaging at 10 h post-injection (**Figure 1A**). In contrast, polystyrene beads injected into the insect hemocoel were encapsulated rapidly (**Figure 1B**). Approximately 80% of the beads were encapsulated within 30 min after injection, and nearly all beads were encapsulated by 1 h. At 2 h post-injection, beads were almost completely covered by haemocytes, and the proportion of encapsulated targets was significantly higher for beads than for nematodes at the same time point (Fisher’s exact test, P < 0.01) (**Figure 1B**).

Before analysing transcriptomic responses, we examined two factors that could influence theinterpretation of bulk RNA-seq data: changes in haemocyte abundance/composition and possible bacterial carryover from nematode preparation. Total haemocyte number was reduced in all injected groups at 2 h, with the strongest decrease observed in the nematode-injected group. Although an early shift in haemocyte composition was suggested, Dirichlet regression did not detect a statistically significant overall effect of condition at either 2 h or 10 h (**Supplementary Figure 1**). By 10 h, total haemocyte number had largely recovered. These results indicate that early changes in haemocyte abundance should be considered when interpreting bulk RNA-seq data, whereas condition-dependent effects on haemocyte composition were not strongly supported by the compositional analysis.

We also assessed whether bacterial contamination from *E. coli* used for nematodeculture remained after bleach treatment. In bacterial growth assays, no colonies were observed on LBagar plates inoculated with the supernatant of bleach-treated nematode suspensions, whereas robust growth was detected in non-treated controls and no growth was detected in M9 buffer controls (**Supplementary Figure 2A**). Consistently, conventional PCR using universal bacterial 16S rRNA gene primers produced anapproximately 500 bp amplicon only in non-treated controls, with no visible band in bleach-treatedsuspensions or M9 buffer controls (**Supplementary Figure 2B**). Together, these results indicate that bacterial carryover was effectively removed by the nematode preparation procedure.

### 3.2 Global transcriptomic structure of haemocyte responses over time

The apparent difference in kinetics between nematodes and beads raises the questions of whetherhaemocytes activate largely similar recognition pathways that proceed more slowly when encounteringa large living organism, or whether distinct recognition mechanisms are engaged in response to nematodes and beads. To distinguish between these possibilities, we performed time-course RNA sequencing of haemocytes following nematode or beads injection to examine dynamic transcriptional changes associated with the initiation of the encapsulation response, as outlined in the experimental schematic (**Supplementary Figure 3**). Approximately 200–300 *C. elegans* individuals or 500–600 beads were injected per insect, and haemolymph containing haemocytes was collected by leg incision at multiple time points (1, 2, 5, and 10 h post-injection).

RNA-seq data were generated from haemocyte-containing haemolymph extracted from insect injected with buffer, polystyrene beads or nematodes at 4 time points with three biological replicates per condition (**Supplementary Table 1**). Sequencing yielded an average of 12.52 million paired-end reads per sample (range: 11.18–14.49 million). Across all samples, an average of 92.16% of bases (range: 90.52–94.04%) achieved a Phred quality score ≥ Q30. Raw reads were inspected for quality and directly aligned to the reference genome, with 70.64%-75.53% mapped to the reference genome. Gene expression was successfully quantified for an average of 9, 313 *M. separata* genes per sample.

**Figure 2:**
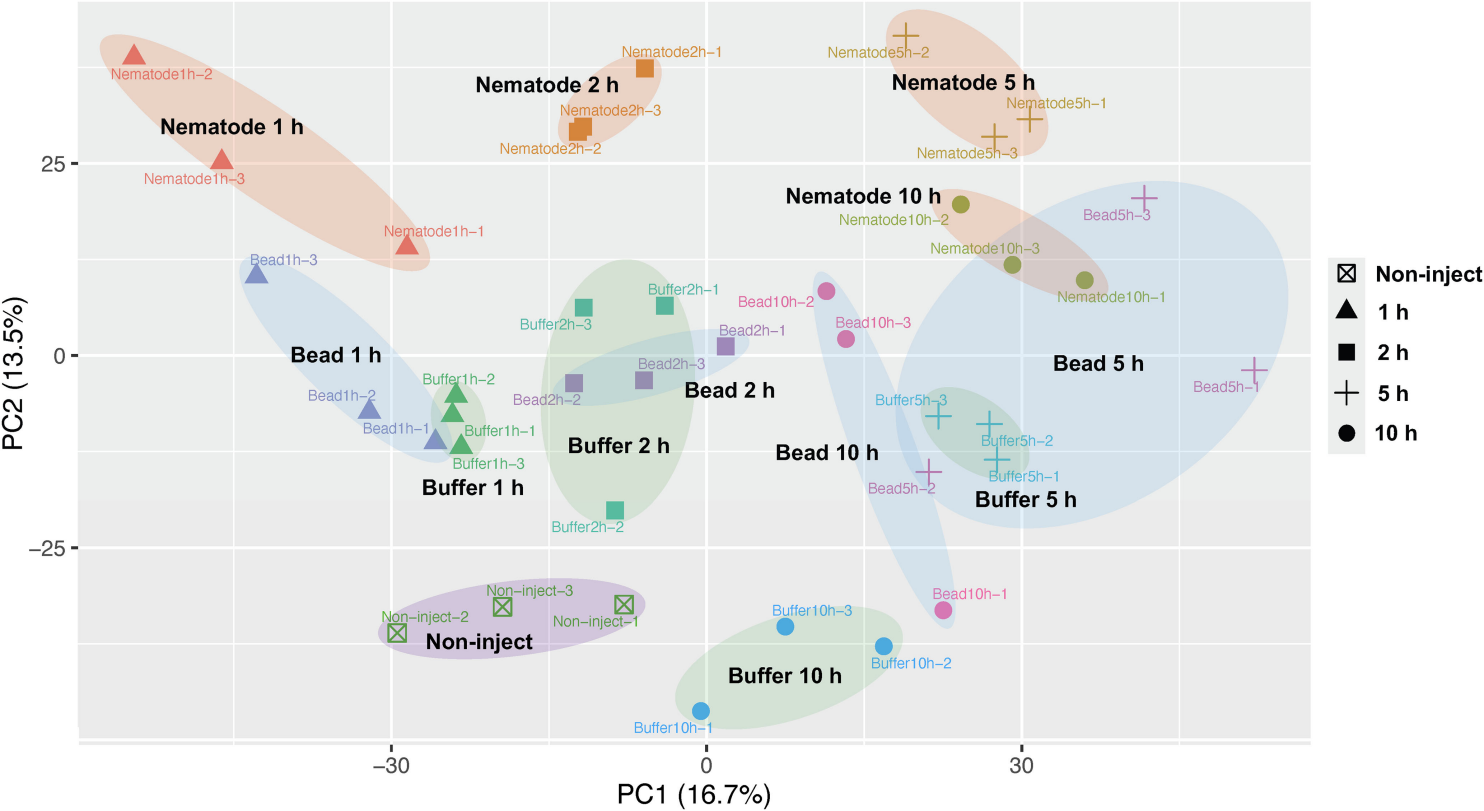
Global transcriptional dynamics of haemocytes following injection stimuli. Principal component analysis (PCA) of haemocyte transcriptomes from non-injected controls and samples injected with buffer, inert beads, or nematodes at 1, 2, 5, and 10 h post-injection. Each point represents one biological replicate, consisting of pooled haemolymph from two insects. Three biological replicates were included per condition per time point. Non-injected controls included three biological replicates (n = 3). Samples are coloured by treatment and time after injection as indicated. Ellipses indicate grouping of replicates within each treatment-time condition. PCA demonstrates temporal separation of transcriptional responses and reveals distinct clustering of nematode-injected samples compared with bead and buffer controls, indicating treatment-specific global transcriptional changes.

To obtain an overview of haemocyte transcriptional responses following nematode injection, we first examined global gene expression patterns using principal component analysis (PCA) (**Figure 2**). PCA revealed a clear separation of samples along the first two principal components, whichexplained 16.7% and 13.5% of the total variance, respectively. Biological replicates clusteredtightly together, indicating good reproducibility of the transcriptomic data. Haemocytes from nematode-injected insects formed a distinct cluster that was clearly separated from bead- and buffer-injected samples as early as 1 h post-injection, suggesting that nematode exposure induces a transcriptional program distinct from responses to inert particles or injection alone. Moreover, nematode-injected samples showed a pronounced temporal trajectory along PC1, with progressive separation between early (1–2 h) and later (5–10 h) time points, indicative of a dynamic and evolving transcriptional response. In contrast, bead- and buffer-injected samples showed partially overlapping distributions at early time points and followed a different temporal pattern from nematode-injected samples. Non-injected controls clustered separately from all injected conditions. Additional PCA plots for PC3 and PC4 are provided in **Supplementary Figure 4** and did not reveal any additional treatment-specific structure beyond that captured by PC1 and PC2. Together, these results indicate that haemocytes mount stimulus-specific transcriptional responses and that nematode injection elicits a distinct and time-dependent global gene expression program.

### 3.3 Time-resolved transcriptional patterns revealed by clustering analysis

**Figure 3:**
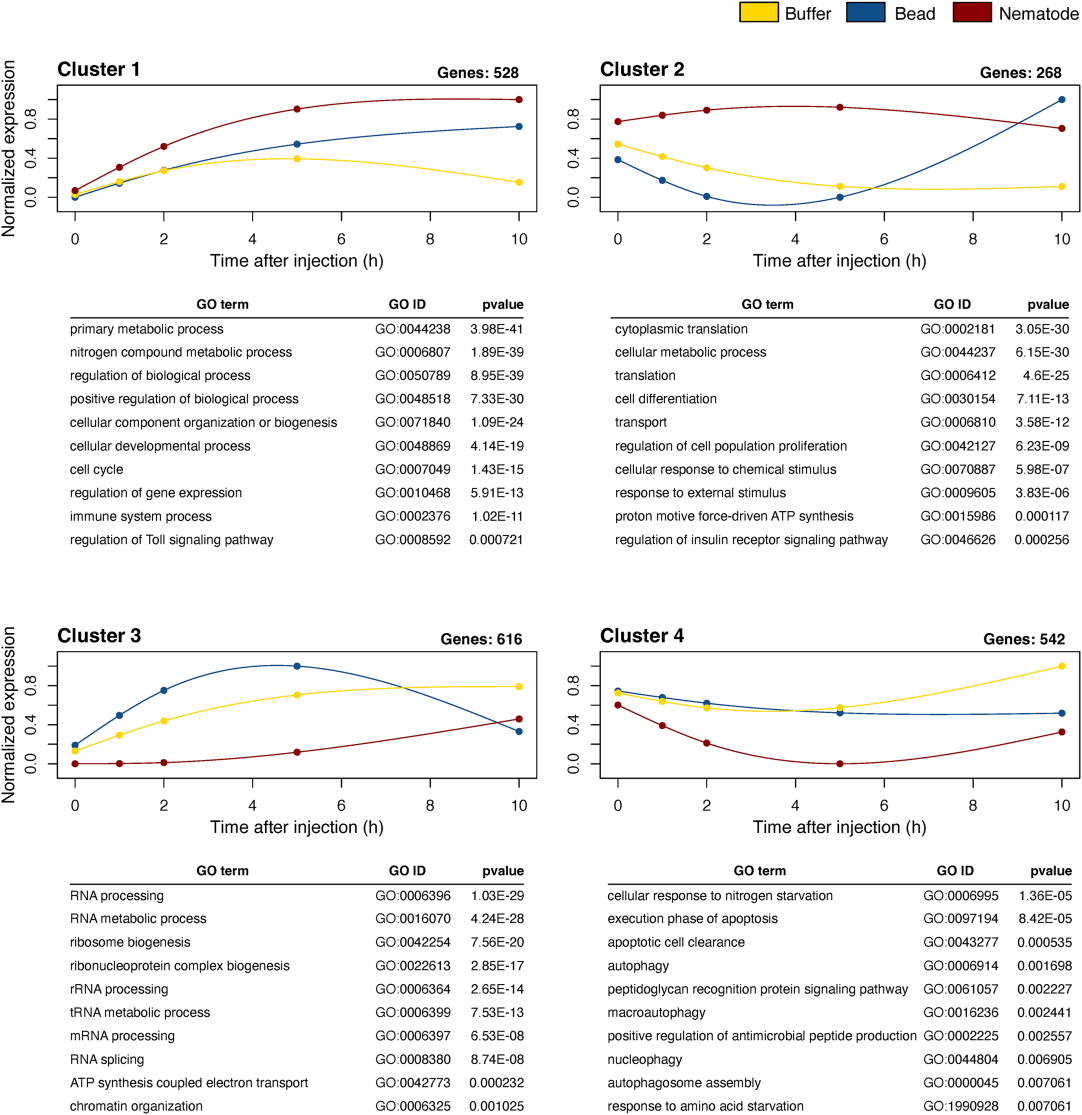
Normalised gene expression trajectories of haemocyte transcripts grouped into four temporal co-expression clusters. Mean expression profiles across time following injection. Expression patterns are shown for each cluster across buffer-, bead-, and nematode-injected samples. Lines represent the average expression of genes assigned to each cluster, with colours indicating treatment (yellow, buffer; blue, bead; red, nematode). The number of genes in each cluster is indicated above each panel. The top 10 enriched Gene Ontology (GO) terms (biological process category) for each cluster are shown below the corresponding plots.

To further characterise the temporal structure of haemocyte transcriptional responses, we performed unsupervised clustering of genes based on their expression dynamics across the time course following injection of nematodes, beads, or buffer (**Figure 3**). Clustering solutions were generated using the spline-based K-means procedure implementedin moanin (55). To determine the optimal number of clusters,we first assessed cluster stability using a resampling-based consensus analysis across K = 2–7 (**Supplementary Figure 5A**). In parallel, we evaluated clustering structure using the elbow method and silhouetteanalysis (**Supplementary Figures 5B, C**) based on distance metrics derived from the expression data. Based on the combined results of these approaches, together with biological interpretability, we selected K = 4 for subsequent analyses.

Using this clustering scheme, genes were grouped into four clusters with distinct temporal expression patterns (**Figure 3**). Cluster 1 comprised genes induced under all conditions, but more strongly in response to nematode injection. Cluster 2 contained genes transiently induced by nematode injection but downregulated in buffer- and bead-injected samples. Cluster 3 showed the opposite tendency, with nematode injection associated with reduced expression relative to the other conditions. Cluster 4 included genes that were broadly downregulated after injection and then partially recovered over time. Together, these patterns indicate that haemocyte transcriptional responses are temporally structured and differ according to the nature of the injected material. In particular, nematode injection was associated with expression trajectories that were not simply delayed versions of the bead- or buffer-induced responses. These clusters therefore provide a useful basis for examining the biological processes associated with stimulus-specific transcriptional dynamics during encapsulation.

To gain insight into the biological processes represented by each temporal expression pattern, we performed Gene Ontology (GO) enrichment analysis for genes within each of the four clusters defined by time-course clustering (**Figure 3**; **Supplementary Tables 2**–**5**). Genes in Cluster 1 were induced following both bead and nematode injection, withsubstantially stronger induction observed after nematode injection, and showed peak expressionaround 4–6 h. These genes were enriched for metabolic and regulatory processes, includingprimary metabolic processes, nitrogen compound metabolism, and regulation of biological processes. Additional enrichment of cellular component organization, developmental processes, and cell cycle–related functions suggests broad changes in cellular activity. Immune-related processes were also enriched, including genes associated with Toll-like signalling components such as a Toll-like receptor and the NF-κB transcription factor Dorsal (**Supplementary Table 6**). These findings indicate that Cluster 1 genes are associated with broad transcriptional and cellular responses, including metabolic and immune-related processes.

Genes in Cluster 2 showed distinct stimulus-dependent dynamics, with transient induction in response to nematode injection and downregulation in buffer- and bead-injected samples at early time points. These genes were enriched for metabolic processes, translational activity, and cellular responses to stimuli. Enriched terms included cytoplasmic translation, ATP synthesis–related processes, and regulation of cell proliferation and differentiation, suggesting increased protein synthesis supported by metabolic activation. Together, these results indicate that Cluster 2 genes are associated with stimulus-dependent activation of translational and metabolic processes.

Genes in Cluster 3 exhibited distinct stimulus-dependent expression patterns, with nematode injection associated with pronounced early downregulation relative to other conditions. These genes were strongly enriched for RNA processing–related functions, including RNA metabolic processes, rRNA and tRNA processing, RNA splicing, and ribosome biogenesis. Additional enrichment of chromatin organization and ATP-related processes suggests coordinated regulation of transcriptional and metabolic activity. Notably, these categories include core components of the gene expression machinery, indicating that nematode injection is associated with a transient suppression of RNA processing and ribosome biogenesis pathways. This pattern is consistent with a reduction in global gene expression capacity during early stages of the haemocyte response.

Genes in Cluster 4 were broadly downregulated following all injection stimuli, with partial recovery over time. These genes were enriched for processes related to autophagy, cellular stress responses, and apoptosis, including macroautophagy, autophagosome assembly, and responses to nutrient deprivation. Immune-related processes were also enriched, including peptidoglycan recognition and regulation of antimicrobial peptide production, together with caspase-related functions. These results suggest that Cluster 4 genes are associated with stress responses, degradation pathways, and immune-related processes.

### 3.4 Time-dependent transcriptional responses induced by buffer injection

**Figure 4:**
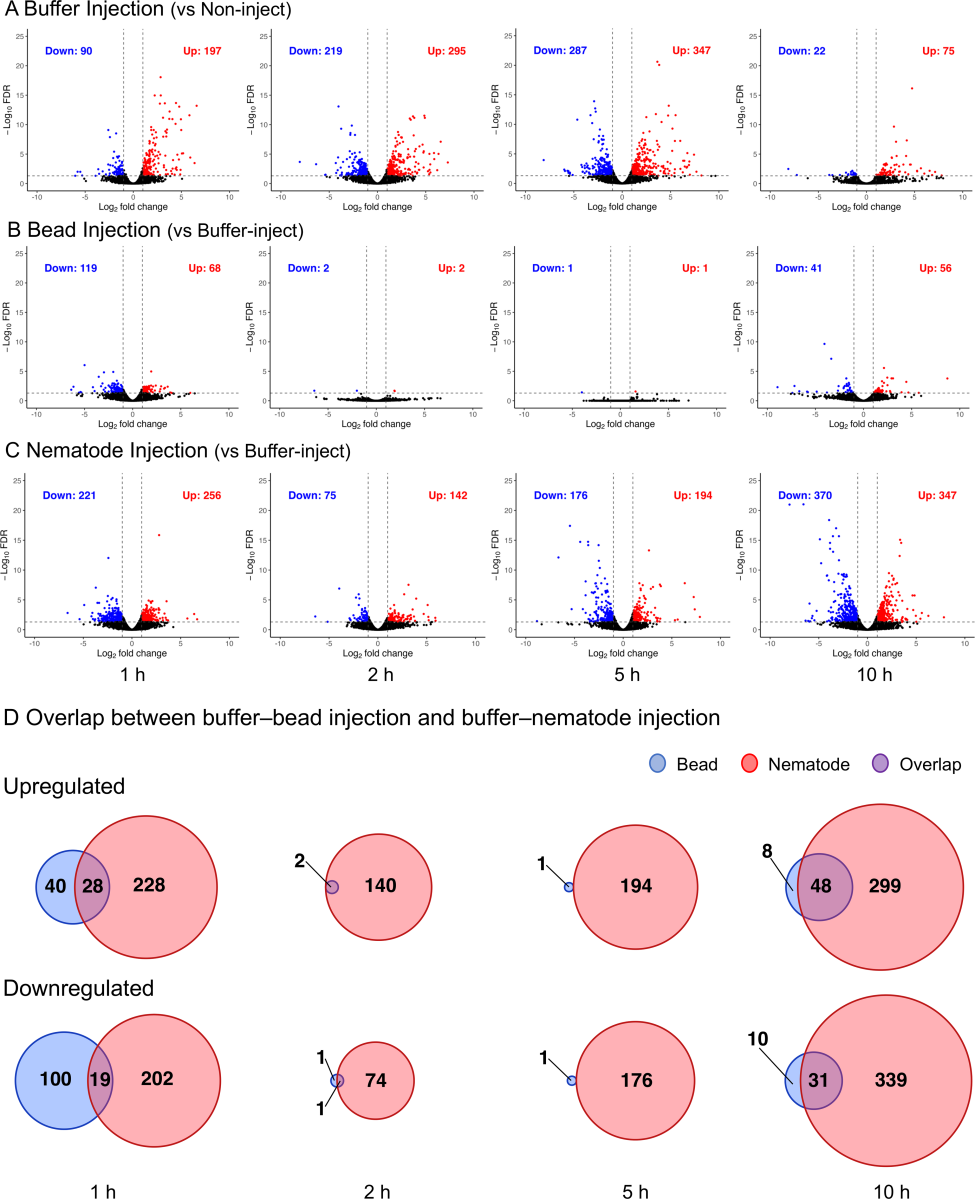
Differential expression analysis of haemocyte transcriptomes. **(A)** Volcano plots showing differentially expressed genes (DEGs) in haemocytes from three pairwise comparisons performed at 1, 2, 5, and 10 h post-injection: **(A)** non-injected controls versus buffer-injected samples, **(B)** buffer-injected controls versus bead injection, and **(C)** buffer-injected controls versus nematode injection. DEGs were defined using a threshold of FDR ≤ 0.05 and |log_2_ fold change| ≥ log_2_(1). **(D)** Venn diagrams showing the overlap of upregulated and downregulated DEGs induced by nematode and bead injection at each time point. Red circles indicate nematode-specific genes, blue circles indicate bead-specific genes, and the purple overlapping region indicates genes shared by both conditions.

To further characterise these responses at the gene level, we next performed differential gene expression analyses. We first examined transcriptional responses induced by buffer injection by comparing buffer-injected samples with non-injected controls at each time point. Buffer injection induced a clear and time-dependent transcriptional response in haemocytes (**Figure 4A**), with distinct functional categories emerging at successive stages following injection (**Table 1**; **Supplementary Table 7**). At 1 h post-injection, genes related to receptor-mediated signalling were significantly enriched, including regulation of transmembrane receptor serine/threonine kinase signaling and cellular responses to transforming growth factor beta stimulus (**Table 1**). Processes associated with cell migration, locomotion, and developmental growth were also enriched, together with regulation of immune response. Enrichment of translation regulation under endoplasmic reticulum stress conditions suggests early activation of stress-responsive protein synthesis pathways. At 2 h post-injection, enriched functions shifted toward cell adhesion and secretory-related processes, including integrin-mediated signaling, cell–matrix adhesion, and platelet degranulation–related pathways (**Table 1**). Additional enrichment of peptide cross-linking and lipid homeostasis suggests remodeling of extracellular and metabolic environments. At 5 h post-injection, translational capacity appeared to be strongly enhanced, as indicated by enrichment of ribosomal large subunit biogenesis and tRNA aminoacylation processes (**Table 1**). At 10 h post-injection, enrichment of meiotic spindle assembly and chromosome segregation regulatory pathways suggests activation of cell cycle–related mechanisms (**Table 1**). Cellular responses to light stimulus were also detected, likely reflecting conserved stimulus-response regulatory modules.

**Table 1:** Representative GO categories enriched in genes upregulated by buffer-injection relative to non-injected controls.

Time	Representative GO ID	Description	Ontology	GO cluster size	Best enrichment p-value
1h	GO:0090092	regulation of transmembrane receptor protein serine/threonine kinase signaling pathway	BP	16	1.60E-06
	GO:0009712	catechol-containing compound metabolic process	BP	6	7.85E-06
	GO:0016477	cell migration	BP	8	2.39E-05
	GO:0071560	cellular response to transforming growth factor beta stimulus	BP	8	3.27E-05
	GO:0060560	developmental growth involved in morphogenesis	BP	26	3.52E-05
	GO:0040011	locomotion	BP	3	4.53E-05
	GO:0050776	regulation of immune response	BP	10	1.03E-04
	GO:0051490	negative regulation of filopodium assembly	BP	1	1.27E-04
	GO:0036493	positive regulation of translation in response to endoplasmic reticulum stress	BP	1	5.59E-04
2h	GO:0002576	platelet degranulation	BP	2	5.43E-07
	GO:0007229	integrin-mediated signaling pathway	BP	12	1.77E-05
	GO:0018149	peptide cross-linking	BP	3	2.34E-05
	GO:0045187	regulation of circadian sleep/wake cycle, sleep	BP	7	2.43E-05
	GO:0007160	cell-matrix adhesion	BP	3	4.17E-05
	GO:0070328	triglyceride homeostasis	BP	3	7.94E-05
	GO:0007475	apposition of dorsal and ventral imaginal disc-derived wing surfaces	BP	3	5.65E-04
5h	GO:0042273	ribosomal large subunit biogenesis	BP	4	5.79E-08
	GO:0006418	tRNA aminoacylation for protein translation	BP	8	1.08E-06
10h	GO:0051257	meiotic spindle midzone assembly	BP	22	6.34E-07
	GO:0051983	regulation of chromosome segregation	BP	13	3.06E-05
	GO:0071482	cellular response to light stimulus	BP	1	4.19E-04

GO enrichment results were summarised using semantic similarity clustering implemented in custom scripts integrating rrvgo and GOSemSim algorithms. Semantic similarity was calculated using the Wang method based on GO hierarchical relationships. Representative GO terms were selected based on the most statistically significant enrichment p-value within each cluster. GO cluster size represents the number of GO terms grouped by semantic similarity.

Although numerous genes were downregulated at early and intermediate time points (90 genes at 1 h, 219 at 2 h, 287 at 5 h, and 22 at 10 h) (**Figure 4A**), GO enrichment analysis revealed relatively weak and dispersed functional signals. Enrichedcategories were largely associated with broad metabolic and signalling pathways, including GPCR signaling and amino acid metabolism, rather than coherent immune-specific programs (**Supplementary Table 7**).

Together, these results demonstrate that buffer injection alone induces a structured, multi-phase transcriptional response in haemocytes, progressing from early signalling and cellular mobilisation, through extracellular and translational adjustment, to late cell cycle– and cytoskeleton-associated programs. Importantly, this response represents a non-specific physiological baseline that must be considered when interpreting transcriptional changes induced by biologically active stimuli.

### 3.5 Limited overlap between buffer–bead and buffer–nematode transcriptional responses

Using the buffer-induced transcriptional response as a reference, we next examined whether bead and nematode injection share common transcriptional reaction. We compared genes differentially expressed in buffer–bead and buffer–nematode contrasts at 1, 2, 5, and 10 h post injection (**Figures 4B, C**) and found that only a small subset of genes (0% to 13.5%) was commonly upregulated or downregulated in bead and nematode injections (**Figure 4D**; **Table 2**).

**Table 2:** Representative GO categories enriched in genes upregulated in haemocytes following nematode-injection relative to bead-injection.

Time	Representative GO ID	Description	Ontology	GO cluster size	Best Enrichment p-value
1h	GO:0003002	regionalization	BP	69	1.29E-08
	GO:0010628	positive regulation of gene expression	BP	22	5.33E-07
	GO:0048523	negative regulation of cellular process	BP	15	7.00E-07
	GO:0050793	regulation of developmental process	BP	24	7.25E-07
	GO:0009966	regulation of signal transduction	BP	8	1.86E-06
	GO:0031323	regulation of cellular metabolic process	BP	35	1.92E-06
	GO:0043170	macromolecule metabolic process	BP	8	6.30E-06
	GO:0048699	generation of neurons	BP	13	1.92E-05
	GO:0045886	negative regulation of synaptic assembly at neuromuscular junction	BP	9	3.26E-05
2h	GO:0035206	regulation of hemocyte proliferation	BP	12	3.18E-08
	GO:0048731	system development	BP	28	6.35E-08
	GO:0051239	regulation of multicellular organismal process	BP	23	7.37E-08
	GO:0007623	circadian rhythm	BP	8	1.45E-07
	GO:0032501	multicellular organismal process	BP	7	5.27E-07
	GO:0048468	cell development	BP	32	5.46E-07
	GO:0042127	regulation of cell population proliferation	BP	12	1.10E-06
	GO:0009605	response to external stimulus	BP	13	1.39E-06
	GO:1902680	positive regulation of RNA biosynthetic process	BP	15	1.45E-05
	GO:0031328	positive regulation of cellular biosynthetic process	BP	16	2.90E-05
	GO:0019953	sexual reproduction	BP	6	8.60E-05
5h	GO:0042418	epinephrine biosynthetic process	BP	2	7.77E-04
10h	GO:0008063	Toll signaling pathway	BP	4	8.07E-05
	GO:0006820	monoatomic anion transport	BP	1	3.89E-04

GO enrichment results were summarised using semantic similarity clustering implemented in custom scripts integrating rrvgo and GOSemSim algorithms. Semantic similarity was calculated using the Wang method based on GO hierarchical relationships. Representative GO terms were selected based on the most statistically significant enrichment p-value within each cluster. GO cluster size represents the number of GO terms grouped by semantic similarity.

At 1 h post-injection, both treatments induced a small overlapping set of differentiallyexpressed genes (28 genes; 9.5%), including genes involved in ion homeostasis and membraneregulatory functions (**Supplementary Table 8**). At 2 h and 5 h, bead injection produced very few transcriptional changes relative to buffer, whereas nematode injection continued to induce robust gene expression responses, resulting in minimal overlap between treatments. Increased variability among 5 h bead-injected samples likely contributed to reduced detection of differentially expressed genes (**Figure 2**). At 10 h, both bead and nematode injection again produced detectable transcriptionalchanges, with modest overlap (48 genes; 13.5%). These shared genes were primarily associated withcellular maintenance and homeostatic processes, including DNA repair, metabolic regulation, and haemocyte differentiation (**Supplementary Table 8**). Together, these results indicate that most transcriptional responses induced by nematode injection cannot be explained solely by physical stimulation or the presence of inert foreign material.

### 3.6 Nematode-specific transcriptional programs relative to bead injection

**Figure 5:**
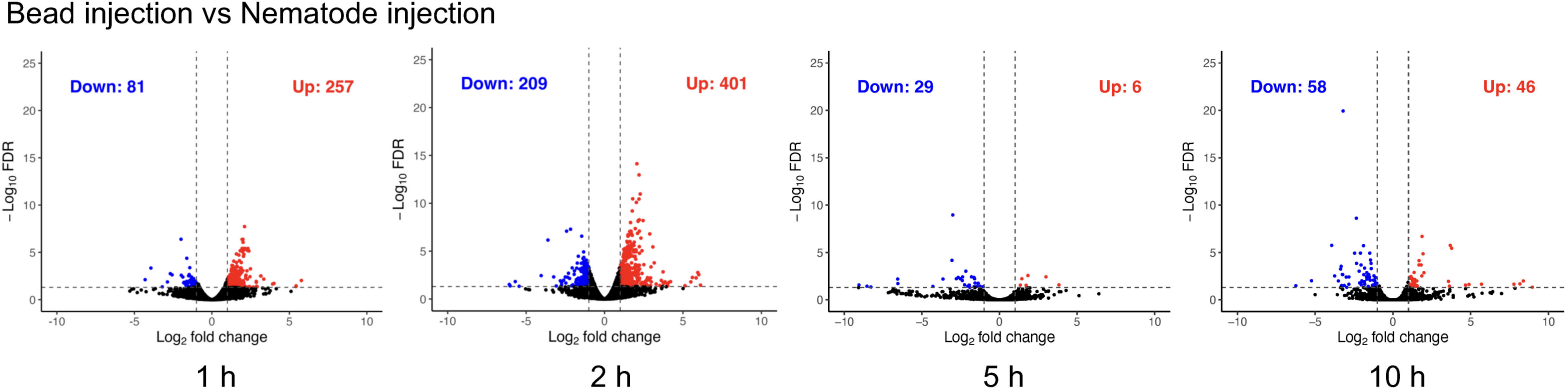
Differential expression analysis of haemocytes nematode-injection versus bead-injection. Volcano plots showing differentially expressed genes (DEGs) in haemocytes from three pairwise comparisons performed at 1, 2, 5, and 10 h post-injection. DEGs were defined using a threshold of FDR ≤ 0.05 and |log_2_ fold change| ≥ log_2_(1).

To directly assess transcriptional differences between responses to inert particles and living organisms, we compared nematode- and bead-injected samples at each time point (**Figure 5**).

At 1 h post-injection, nematode-injection induced a distinct and coherent set of upregulated genes relative to bead injection (**Table 2**; **Supplementary Table 9**). GO enrichment analysis showed strong overrepresentation of regulatory and developmentalpatterning processes, including regionalisation, regulation of gene expression, developmentalregulation, and signal transduction. Enrichment of cellular and macromolecule metabolic regulationfurther suggests rapid transcriptional and metabolic reprogramming. GO terms related to neuronal or synaptic processes were also enriched; however, inspection of the underlying gene sets indicated that these annotations primarily reflect broadly conserved regulatory factors, such as kinases (Abl, PKC), mTOR-related components (Raptor), Hippo pathway regulators (Mob1), and RNA-binding proteins (FXR1-like), rather than neuron-specific genes (**Supplementary Table 10**). Together, these results indicate that haemocytes initiate an early transcriptional response to nematode challenge that differs qualitatively from that induced by inert particles. At 2 h post-injection, transcriptional differences between nematode- and bead-injection remained pronounced. Genes upregulated following nematode injection were enriched for regulation of hemocyte proliferation, cell development, and multicellular regulatory processes, together with responses to external stimuli and positive regulation of RNA biosynthesis. These enrichments suggest continued cellular activation and biosynthetic priming in response to biological material (**Table 2**; **Supplementary Table 9**). At 5 h, only a small number of DE genes were detected (**Figure 5**), which probably reflect the high variability among bead-injected samples at this time point (**Figure 2**). By 10 h post-injection, enrichment shifted toward innate immune and transport-related functions. The Toll signalling pathway was significantly enriched, together with monoatomic anion transport (**Table 2**; **Supplementary Table 9**), suggesting later-stage activation of immune regulatory and cellular homeostasis pathways.

Together, these results indicate that haemocytes respond to nematode-injection through temporally dynamic transcriptional programs, progressing from early regulatory reprogramming to later immune-associated signalling responses, which are largely absent following inert bead injection.

### 3.7 Biased and coordinated regulation of the Toll signalling pathway

**Figure 6:**
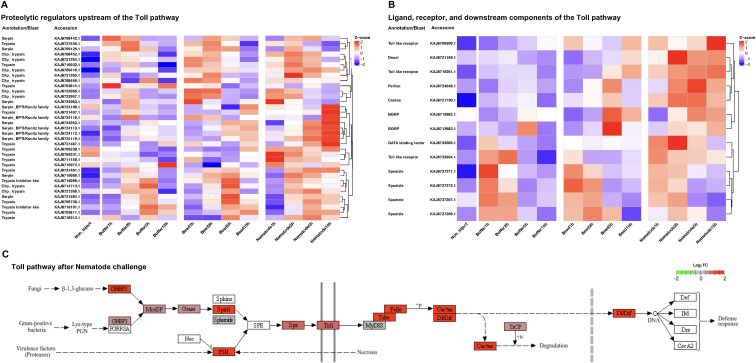
Nematode-induced transcriptional changes in the toll signalling pathway heatmaps showing the expression dynamics of a core toll signalling gene set across buffer-, bead-, and nematode-injected samples. Genes are grouped into **(A)** upstream components including serine proteases, pattern-recognition–related genes, and ligands, and **(B)** downstream signalling components, including regulatory factors and transcriptional effectors. Expression values represent mean TPM values averaged across three biological replicates and are z-score normalised per gene to visualise relative temporal expression patterns following injection. **(C)** KEGG pathway map of the Toll signalling pathway illustrating transcriptional changes 2 h after nematode injection. Log2 fold change were overlaid onto the pathway diagram to visualise nematode-associated regulation of Toll pathway components at this time point. When multiple genes mapped to the same KEGG Orthology (KO), their log2 fold change values were averaged. Boxes are coloured according to log2 fold change, where red indicates upregulation and green indicates downregulation, and colour intensity reflects the magnitude of expression change. The colour scale was fixed from −2 to +2, with values outside this range capped.

To characterise immune-related transcriptional responses, we examined the expression dynamics of a curated core Toll signalling gene set identified from differential expression analyses across buffer-, bead-, and nematode-injected samples (**Figure 6**). This approach enabled pathway-level interpretation while retaining temporal resolution.

Analysis of upstream Toll pathway components revealed that several genes responded broadly to injection. CLIP-domain serine proteases (56) and serine protease inhibitors (57)showed induction following both bead and nematode injection, particularly at early time points, indicating that activation of upstream proteolytic cascades is not specific to nematode exposure. Similarly, a subset of pattern-recognition–related genes, including β-1, 3-glucan recognition protein-like genes (58), exhibited increased expression after bead and nematode injection, consistent with a shared response to tissue perturbation and foreign material.

Despite this shared upstream responsiveness, nematode injection was associated with distinct quantitative and temporal features within the Toll pathway. Genes encoding the Toll ligand Spätzle (59)and several Toll-like receptors displayed higher expression levels and reduced temporal fluctuation in nematode-injected samples compared with bead-injected controls, particularly at later time points (**Figure 6A**). This pattern suggests that nematode injection stabilises or sustains Toll pathway–associated transcription rather than inducing a transient response.

Downstream components of the Toll pathway exhibited more heterogeneous expression patterns. Genesencoding signalling regulators such as Pellino, Cactus, and the NF-κB transcription factorDorsal (58) showed increased expression in nematode-injectedsamples. In addition, the antimicrobial peptide Defensin (Galiomicin), a known Toll-responsive effector, was also upregulated following nematode injection (**Supplementary Figure 6**). Similar trends were also detectable, albeit more weakly or transiently, in bead- or buffer-injected samples (**Figure 6B**). These patterns indicate that downstream Toll signalling is modulated in a graded manner and cannot be classified as strictly nematode-specific.

Consistent with gene-level analyses, pathway-level enrichment analysis indicated activation of the Toll signalling pathway following nematode injection (**Figure 6C**). However, enrichment was moderate rather than exclusive, reflecting the partial overlap of Toll-related transcriptional responses across treatments.

Together, these results demonstrate that nematode injection biases the regulation of the Toll signalling pathway toward higher and more temporally stable expression of key components, rather than uniquely activating the pathway. Upstream sensing and downstream signalling elements are engaged to varying degrees by all injection conditions, but nematodes elicit a distinct transcriptional state characterised by sustained and coordinated Toll pathway-associated gene expression.

## 4 Discussion

Understanding how insect immune cells distinguish living multicellular parasites from inert material remains a central problem in host–parasite biology. Here, by explicitly separating transcriptional responses induced by injection, inert beads, and living nematodes, we provide a framework that allows organism-specific host responses to be interpreted without confounding effects of physical perturbation or foreign-body exposure. A critical insight from this framework is that injection establishes a permissive physiological state rather than an immune state. The buffer-induced transcriptional program progresses through multiple regulatory phases, but does not converge on pathways typically associated with pathogen recognition or defence. Instead, it reflects coordinated cellular adjustment and recovery. This finding underscores that injection-related transcriptional activity should not be treated as background noise, but as an organised baseline against which additional biological signals are evaluated.

Nematode exposure consistently shifts haemocytes away from the injection-defined baseline into distinct transcriptional states. Importantly, these states are not simply exaggerated versions of the baseline response or bead-injection. Time-course clustering indicates that nematode-associated DE genes follow independent temporal trajectories, implying early discrimination and engagement of regulatory programs that are qualitatively different from those induced by injection alone or bead-injection. This argues against a model in which nematode responses emerge gradually through accumulated damage signals. The functional character of nematode-associated differential expression further supports this interpretation. Rather than being dominated by generic stress responses, nematode-induced DE preferentially involves regulatory, immune, and membrane-associated processes. The enrichment of Toll pathway components within this broader transcriptional context suggests that immune signalling is embedded within a coordinated response to biological activity, rather than triggered by canonical microbial ligands alone. This distinction is particularly relevant in the context of entomopathogenic nematodes (EPNs), which harbour symbiotic bacteria that strongly influence host immunity. Previous RNA-seq studies using axenic *Steinernema* infections in Drosophila reported early downregulation of Toll pathway components (e.g., GNBP3, Serpin-27A), followed by later induction of antimicrobial peptides and regulatory factors (60). However, even in the absence of symbiotic bacteria, EPN-derived ES products and EV can modulate host immune responses, complicating the interpretation of transcriptional changes (45, 46). In contrast, the non-parasitic nematode *C. elegans* lacks such immunomodulatory mechanisms, allowing a clearer assessment of intrinsic host responses. Consistent with this, our time-course and KEGG analyses suggest that Toll pathway activation may represent an intrinsic component of the host response to nematodes.

Importantly, haemocyte responses to nematodes are not characterised by simple on-off activation of immune pathways. Instead, they reflect biased and temporally stabilised engagement of conserved signalling modules. Although several upstream components of the Toll pathway respond broadly to injection and inert material, nematode exposure is associated with more coherent and sustained regulation across multiple pathway levels. This pattern indicates qualitative differences in pathway engagement rather than strict nematode specificity at the level of individual genes. This point is illustrated by the behaviour of individual pathway-related genes. For example, betaGRP-like genes were upregulated not only after nematode injection but also after bead injection, even though polystyrene beads do not contain β-glucan. In *M. separata*, bead injection has also been reported to induce other recognition- and adhesion-related molecules, including C-type lectins and integrins (26, 28), suggesting that some upstream recognition factors respond broadly to foreign surface cues rather than specifically to nematodes. By contrast, nematode challenge was associated with more consistent regulation across the pathway as a whole, including upregulation of the negative regulator cactus, which is consistent with feedback control within an actively engaged Toll-signalling network. Together, these observations suggest that nematode discrimination is reflected less in the behaviour of any single upstream gene than in the coordinated and sustained engagement of the pathway overall.

This mode of response has important implications for how hosts recognise multicellular parasites. Unlike inert beads, nematodes differ in size, shape, and mechanical properties, actively move within host tissues, and maintain prolonged contact with haemocytes. Rather than activating immune pathways through a single nematode-specific cue, these features are likely to provide persistent and integrated stimuli that reinforce signalling over time, thereby shifting haemocytes into a distinct early transcriptional state. In contrast, at later stages, transcriptional responses showed partial convergence between bead and nematode challenge. Genes associated with DNA repair, metabolic regulation, and haemocyte differentiation were upregulated under both conditions, suggesting engagement of processes related to cellular maintenance and restoration of homeostasis. This transition indicates that, while early responses reflect stimulus-specific recognition, later responses may be shaped by the cumulative effects of sustained interactions with foreign material. Activation of Toll pathway components following challenge with axenically prepared nematodes further supports the idea that canonical innate immune signalling can be engaged by multicellular organisms themselves. This does not imply that nematodes are recognised through the same mechanisms as bacteria or fungi. Rather, our results support a model in which distinct upstream cues converge on shared downstream signalling modules, allowing haemocytes to deploy conserved immune pathways while retaining specificity toward biologically active multicellular targets.

At the same time, our conclusions remain limited to transcriptional evidence. The present study does not establish a causal role for the Toll pathway in nematode recognition or encapsulation, and the relationship between Toll-associated transcription and immune execution therefore remains correlative. In addition, because the analysis is based on bulk haemocyte RNA-seq, it cannot fully resolve transcriptional regulation within individual haemocyte types, although the supplementary haemocyte-counting experiments suggest that later transcriptional differences are not explained solely by shifts in haemocyte composition. Finally, the RNA-seq time course was restricted to 0–10 h, and therefore does not capture later transcriptional states associated with capsule maturation or resolution. These limitations define several clear directions for future work. Functional analyses, such as RNAi-mediated knockdown and targeted assays of Toll pathway activity, will be required to test causality. Cell-resolved approaches, including marker-based deconvolution or single-cell RNA-seq, will help determine how transcriptional regulation is distributed across haemocyte types. In addition, comparisons between live and dead nematodes, or between motile and immobilised animals, should help distinguish the contributions of movement, prolonged contact, and other biological cues. In this context, genetically tractable nematodes such as *C. elegans* provide a useful system for dissecting how multicellular organisms are recognised by insect haemocytes. Overall, by distinguishing injection-induced, inert particle-induced, and nematode-associated transcriptional responses, this study shows that haemocytes respond selectively to living multicellular targets and that this recognition cannot be explained by physical presence alone.

## Data Availability

The datasets presented in this study can be found in online repositories. The names of the repository/repositories and accession number(s) can be found below: https://www.ddbj.nig.ac.jp/, PRJDB40401.

## Supplementary material

Supplementary Figure 1 Haemocyte composition across treatments. Relative proportions of haemocyte types in non-injected, buffer-, bead-, and nematode-injected samples at 2 and 10 h post-treatment. Haemocytes were classified into granulocytes, plasmatocytes, and other cell types based on morphological characteristics.

Supplementary Figure 2 Assessment of bacterial carryover in samples used for RNA-seq-related analyses. **(A)** Representative agar plates showing microbial growth in samples a-c. Visible colony growth was detected in sample a, whereas little or no growth was observed in samples b and c. **(B)** Representative gel image of PCR-based detection from the corresponding samples, showing a prominent band in lane a and no comparable amplification in lanes b or c. Marker, DNA size marker.

Supplementary Figure 3 Schematic overview of the experimental workflow. Polystyrene beads or nematodes were injected into the insect hemocoel, after which haemolymph containing haemocytes was collected by leg incision at the indicated time points. RNA was extracted from the collected haemocyte-containing haemolymph and subjected to RNA-seq analysis. (created with BioRender.com).

Supplementary Figure 4 Additional principal component analysis of RNA-seq samples. Principal component analysis showing sample distribution along PC3 and PC4. Samples are colored by treatment group and symbol-coded by time point. In contrast to PC1 and PC2, PC3 and PC4 did not reveal additional clustering patterns, indicating that the major structure of sample-to-sample variation was captured primarily by the first two principal components.

Supplementary Figure 5 Evaluation of cluster number for k-means clustering. (A) Cluster assignment matrices obtained from k-means clustering across different numbers of clusters (K = 2-7). (B) Elbow plot showing the total within-cluster sum of squares across K values. (C) Silhouette plot showing the average silhouette width across K values. Together, these analyses were used to evaluate the appropriate number of clusters for downstream classification of expression patterns.

Supplementary Figure 6 Expression patterns of antimicrobial peptide-related genes across treatments and time points. Heat map showing the relative expression patterns of selected antimicrobial peptide-related genes across non-injected, buffer-, bead-, and nematode-treated samples over the time course. Gene names and corresponding locus IDs are shown on the left. Colors indicate z-score-normalised expression values, with red indicating relatively high expression and blue indicating relatively low expression. The dendrogram on the right shows hierarchical clustering based on similarity in expression profiles among genes.

Supplementary Table 1 Summary of RNA-seq mapping and quantification statistics for all samples.

Supplementary Tables 2-5 Enriched GO terms in genes assigned to each time-course expression cluster.

Supplementary Table 6 Lists of genes assigned to each time-course expression cluster.

Supplementary Table 7 GO terms significantly upregulated or downregulated by buffer injection.

Supplementary Table 8 Genes commonly up/down-regulated between bead- and nematode-injection.

Supplementary Table 9 Enriched GO terms in differentially expressed genes in response to nematode versus bead injection.

Supplementary Table 10 Lists of genes differentially expressed between nematode- and bead-injection.
