# Correction: Time-resolved transcriptomics of haemocyte discrimination between challenges by nematodes and inert material in the Oriental Armyworm *Mythimna separata*

**DOI:** 10.3389/fimmu.2026.1870895

**Published:** 2026-05-12

**Authors:** Masaya Ono, Xiaolan Li, Yasunobu Maeda, Rei Nishimura, Jion Tsuruta, Akemi Yoshida, Kazuki Sato, Taisei Kikuchi

**Affiliations:** 1 Laboratory of Parasite Systems Biology, Department of Integrated Biosciences, Graduate School of Frontier Sciences, The University of Tokyo, Kashiwa, Chiba, Japan; 2 Department of infectious disease, Faculty of Medicine, The University of Miyazaki, Miyazaki, Japan; 3 Genomics and Bioenvironmental Science, Frontier Science Research Center, University of Miyazaki, Miyazaki, Japan

**Keywords:** encapsulation, haemocyte, immunity, insect, nematode, RNA-seq

Author “Xiaolan Li” was erroneously written as “Li Xiaolan”.

The last name is Li and the first name is Xiaolan.

In addition, the author’s name was incorrectly reflected in the citation and copyright statement.

The initials used for this author in the **Author contributions** section were also incorrect “LX” instead of “XL”, and have now been corrected.

The original version of this article has been updated.

