# Supplementary figures and images for: Time-resolved transcriptomics of haemocyte discrimination between challenges by nematodes and inert material in the oriental armyworm Mythimna separata
Source: Front Immunol. 2026 Apr 29;17:1810925. doi: 10.3389/fimmu.2026.1810925 (PMC13206333; doi:10.3389/fimmu.2026.1810925)

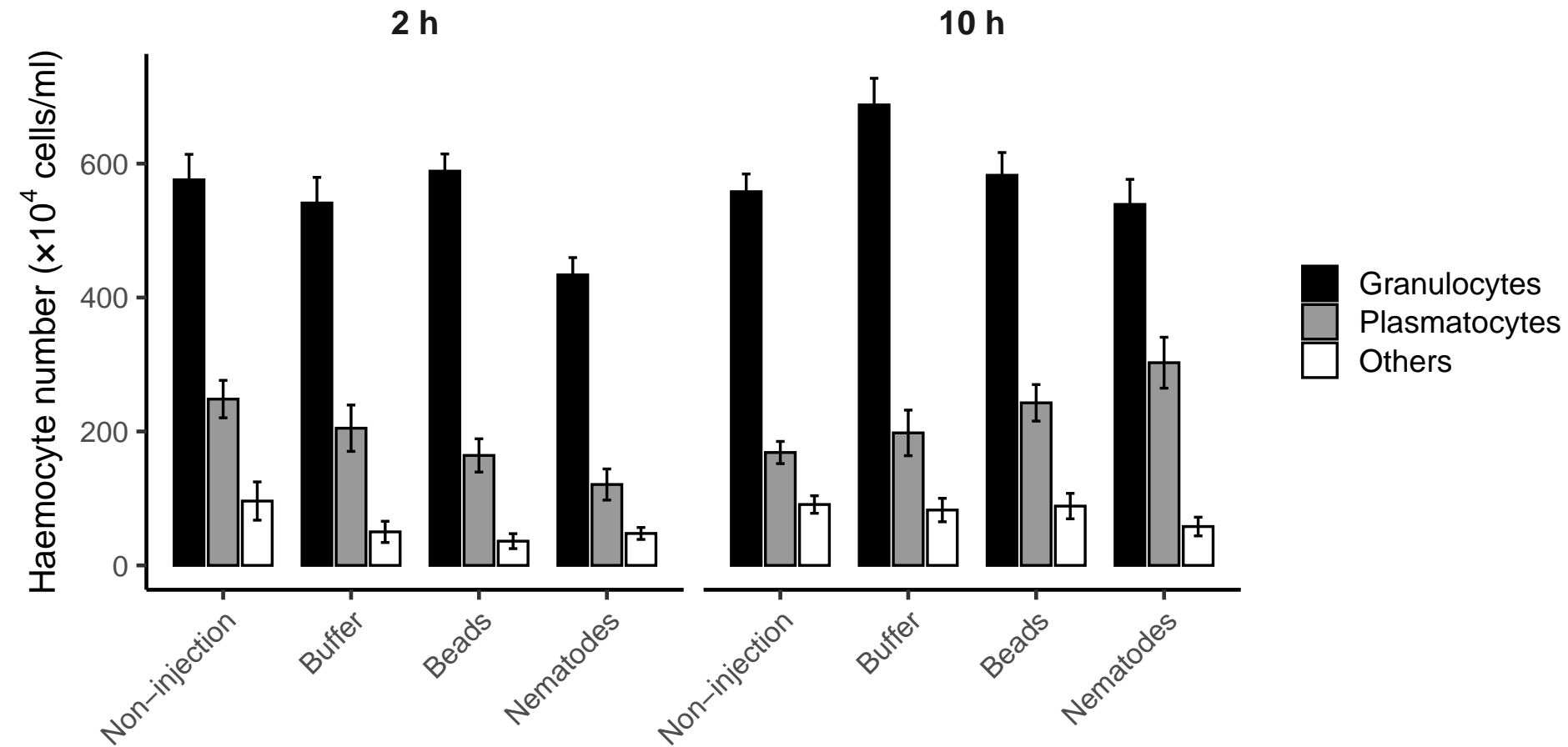

Supplement: Supplementary Figure 1 — Haemocyte composition across treatments. Relative proportions of haemocyte types in non-injected, buffer-, bead-, and nematode-injected samples at 2 and 10 h post-treatment. Haemocytes were classified into granulocytes, plasmatocytes, and other cell types based on morphological characteristics. [file DataSheet1.pdf]

**A**

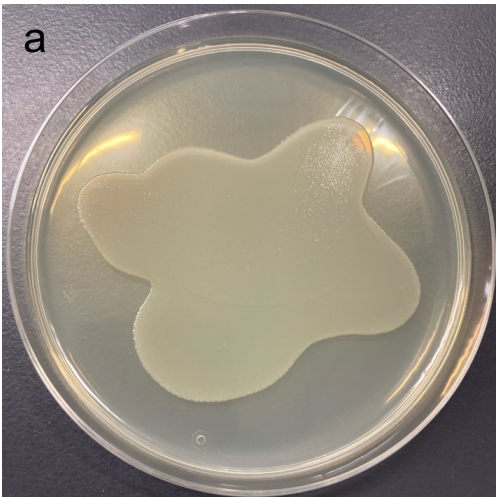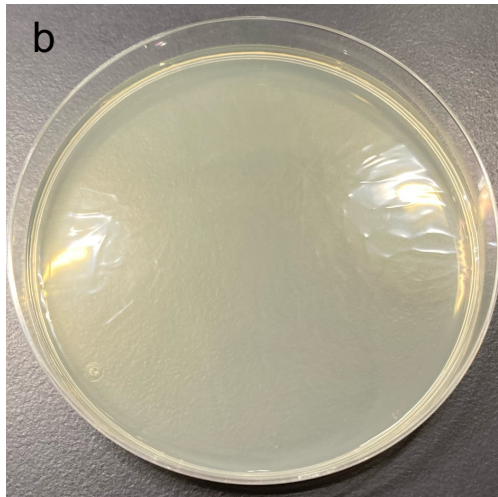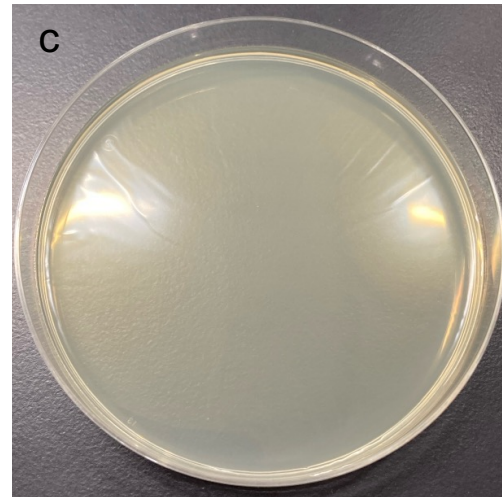

**B**

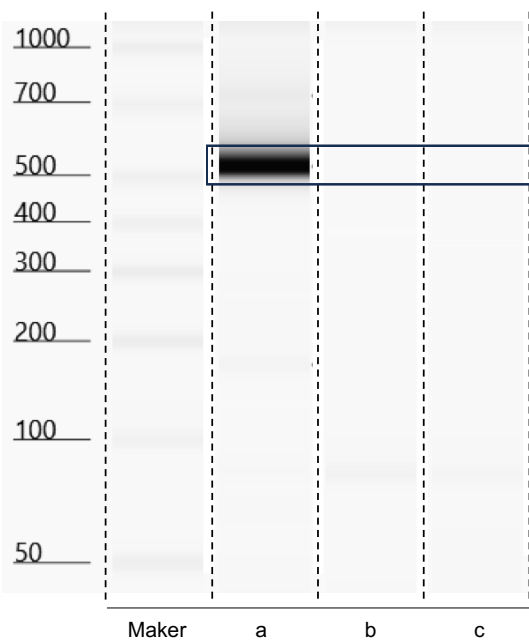

Supplement: Supplementary Figure 2 — Assessment of bacterial carryover in samples used for RNA-seq-related analyses. (A) Representative agar plates showing microbial growth in samples a-c. Visible colony growth was detected in sample a, whereas little or no growth was observed in samples b and c. (B) Representative gel image of PCR-based detection from the corresponding samples, showing a prominent band in lane a and no comparable amplification in lanes b or c. Marker, DNA size marker. [file DataSheet2.pdf]

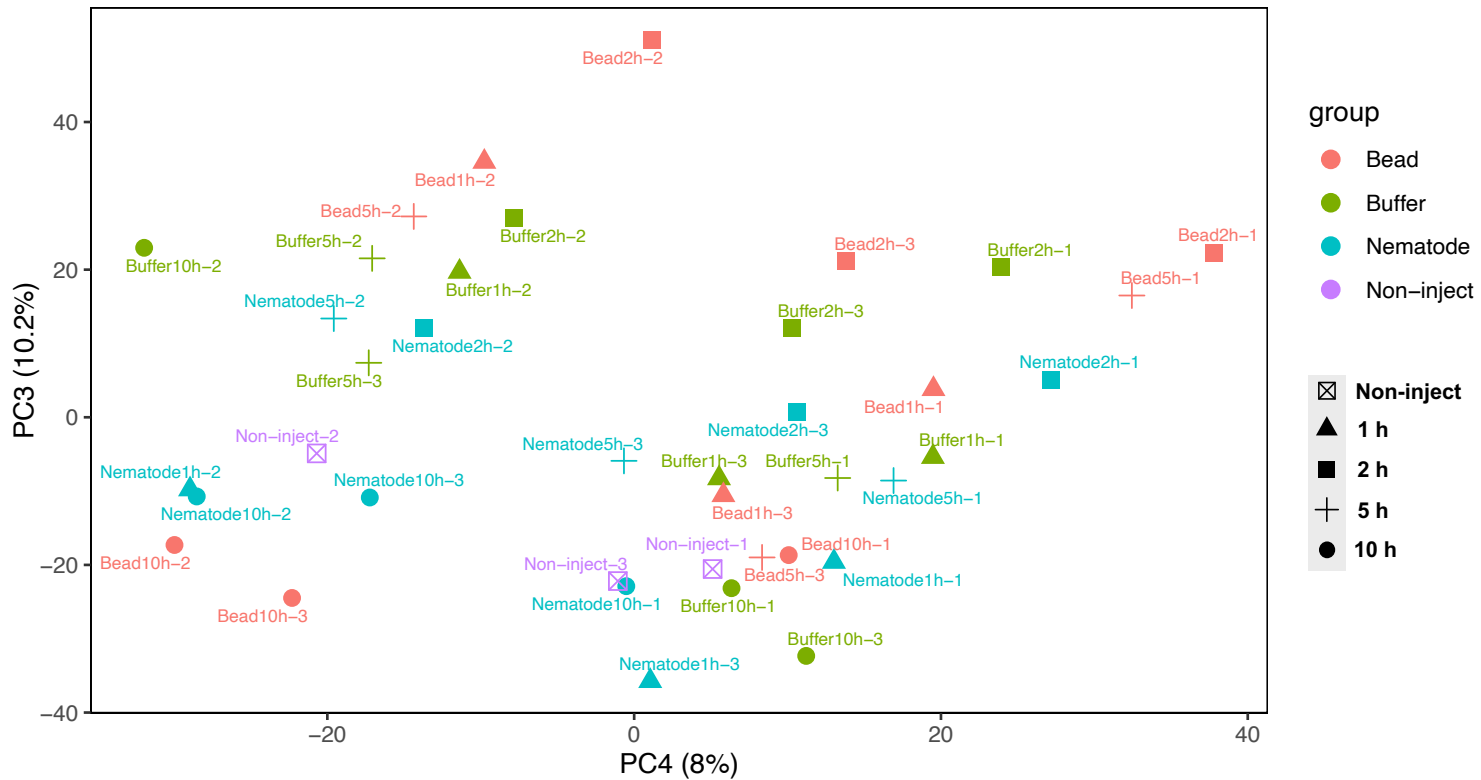

Supplement: Supplementary Figure 4 — Additional principal component analysis of RNA-seq samples. Principal component analysis showing sample distribution along PC3 and PC4. Samples are colored by treatment group and symbol-coded by time point. In contrast to PC1 and PC2, PC3 and PC4 did not reveal additional clustering patterns, indicating that the major structure of sample-to-sample variation was captured primarily by the first two principal components. [file DataSheet4.pdf]

**A Kmeans plot**

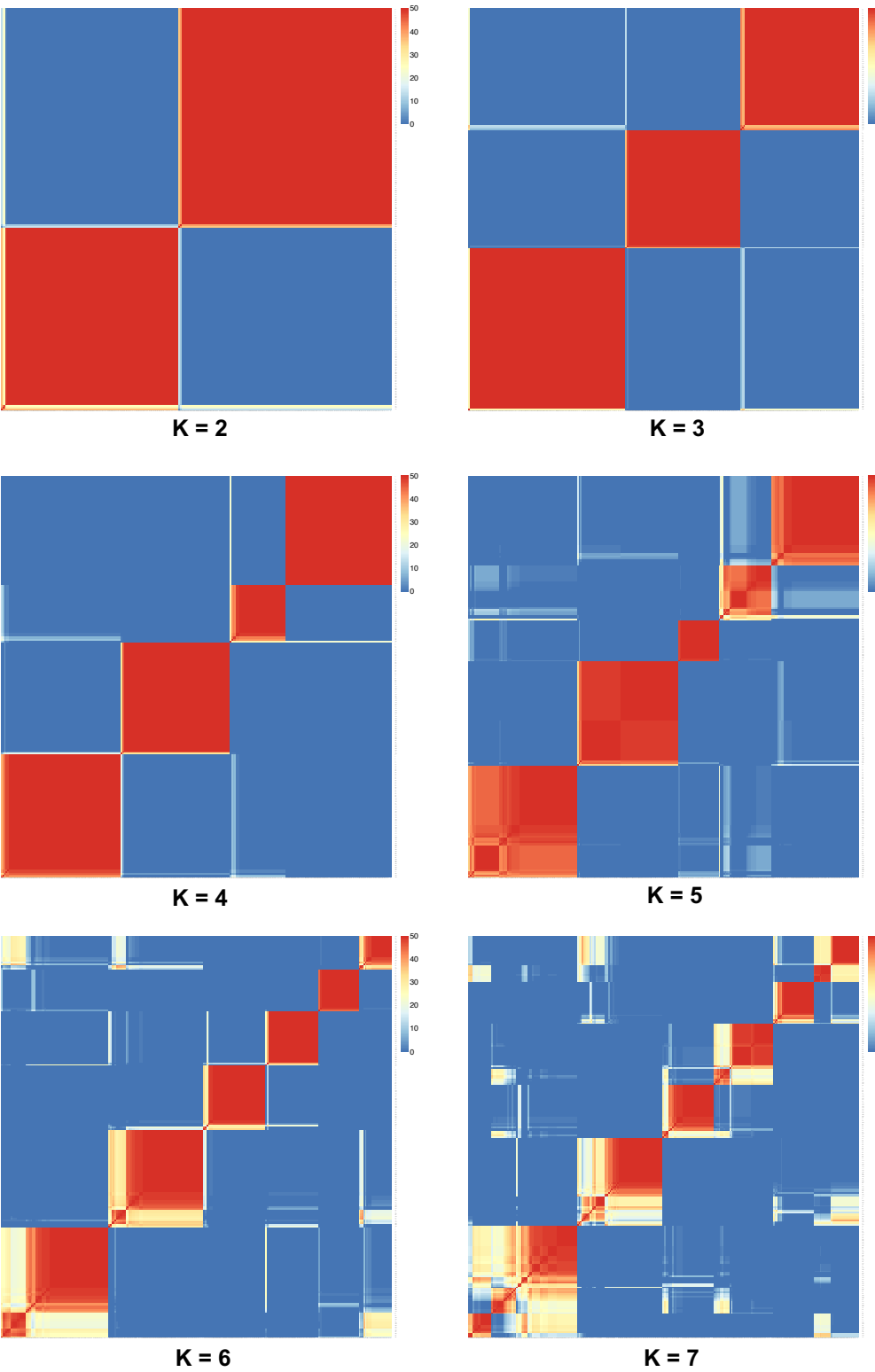

**B Elbow plot**

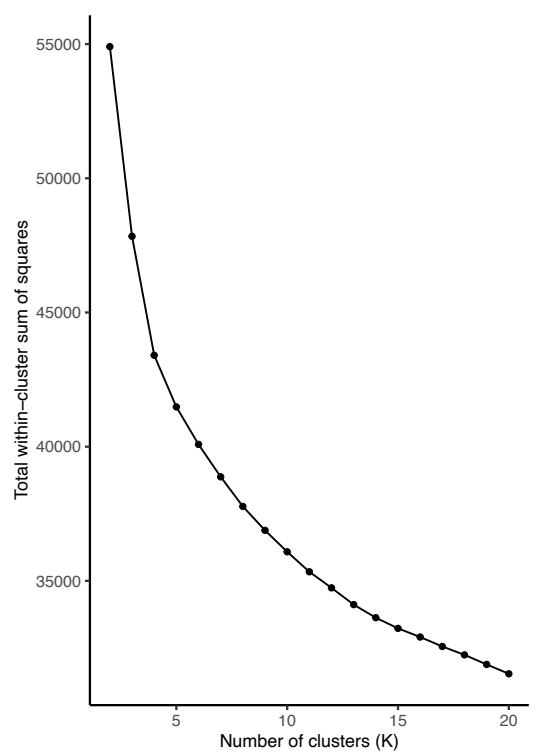

**C Silhouette plot**

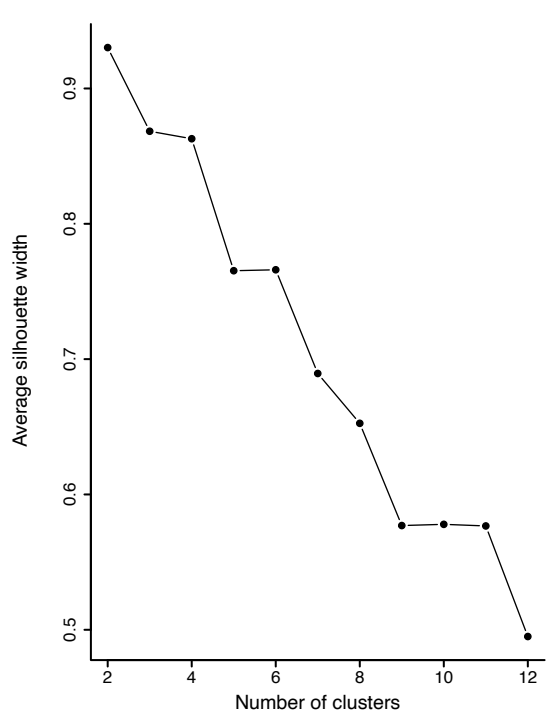

Supplement: Supplementary Figure 5 — Evaluation of cluster number for k-means clustering. (A) Cluster assignment matrices obtained from k-means clustering across different numbers of clusters (K = 2-7). (B) Elbow plot showing the total within-cluster sum of squares across K values. (C) Silhouette plot showing the average silhouette width across K values. Together, these analyses were used to evaluate the appropriate number of clusters for downstream classification of expression patterns. [file DataSheet5.pdf]

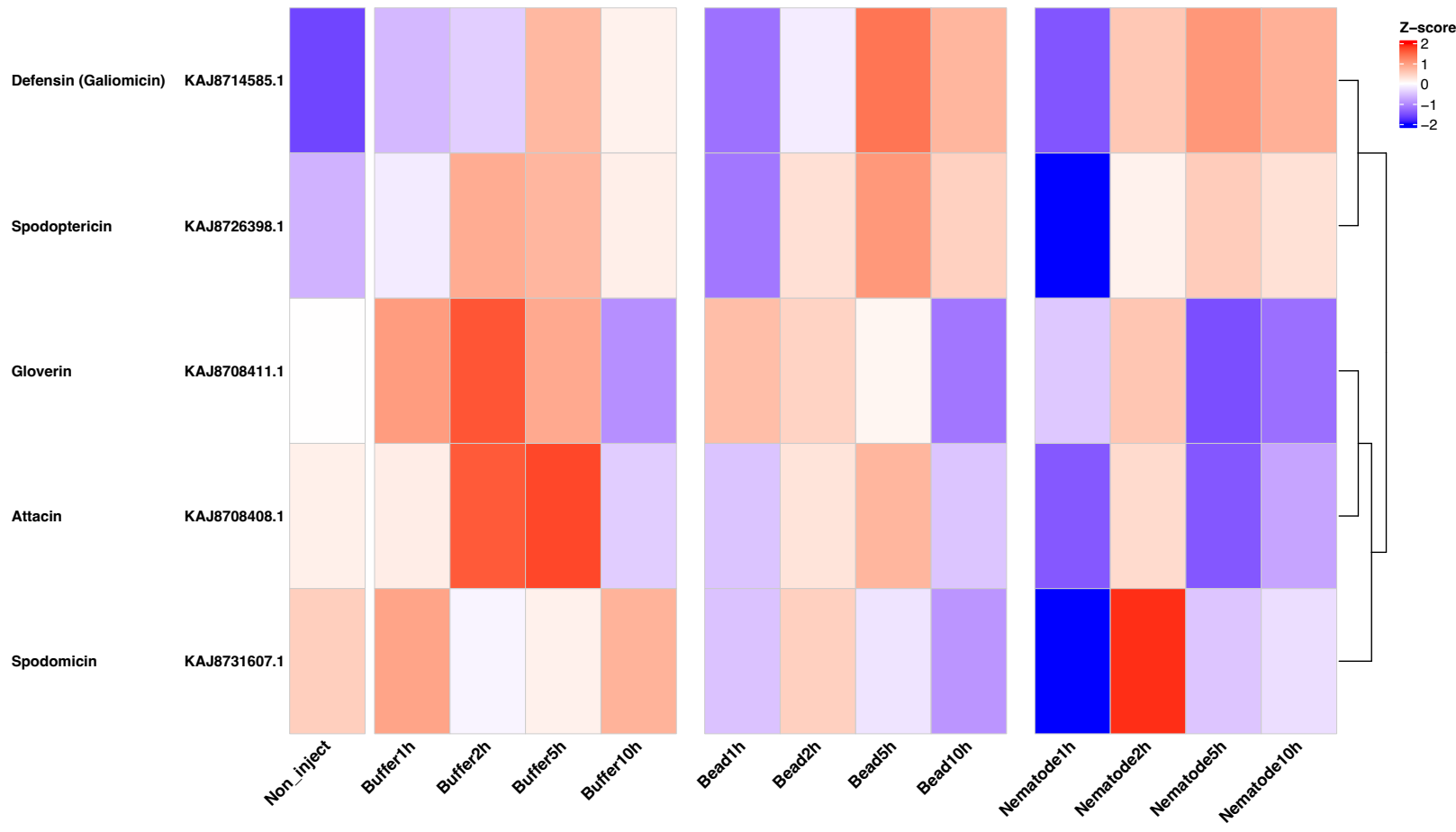

Supplement: Supplementary Figure 6 — Expression patterns of antimicrobial peptide-related genes across treatments and time points. Heat map showing the relative expression patterns of selected antimicrobial peptide-related genes across non-injected, buffer-, bead-, and nematode-treated samples over the time course. Gene names and corresponding locus IDs are shown on the left. Colors indicate z-score-normalised expression values, with red indicating relatively high expression and blue indicating relatively low expression. The dendrogram on the right shows hierarchical clustering based on similarity in expression profiles among genes. [file DataSheet6.pdf]
