# Supplementary material for: Time-resolved transcriptomics of haemocyte discrimination between challenges by nematodes and inert material in the oriental armyworm Mythimna separata
Source: Front Immunol. 2026 Apr 29;17:1810925. doi: 10.3389/fimmu.2026.1810925 (PMC13206333; doi:10.3389/fimmu.2026.1810925)
Supplement: Supplementary Figure 3 — Schematic overview of the experimental workflow. Polystyrene beads or nematodes were injected into the insect hemocoel, after which haemolymph containing haemocytes was collected by leg incision at the indicated time points. RNA was extracted from the collected haemocyte-containing haemolymph and subjected to RNA-seq analysis. (created with BioRender.com). [file DataSheet3.pdf]

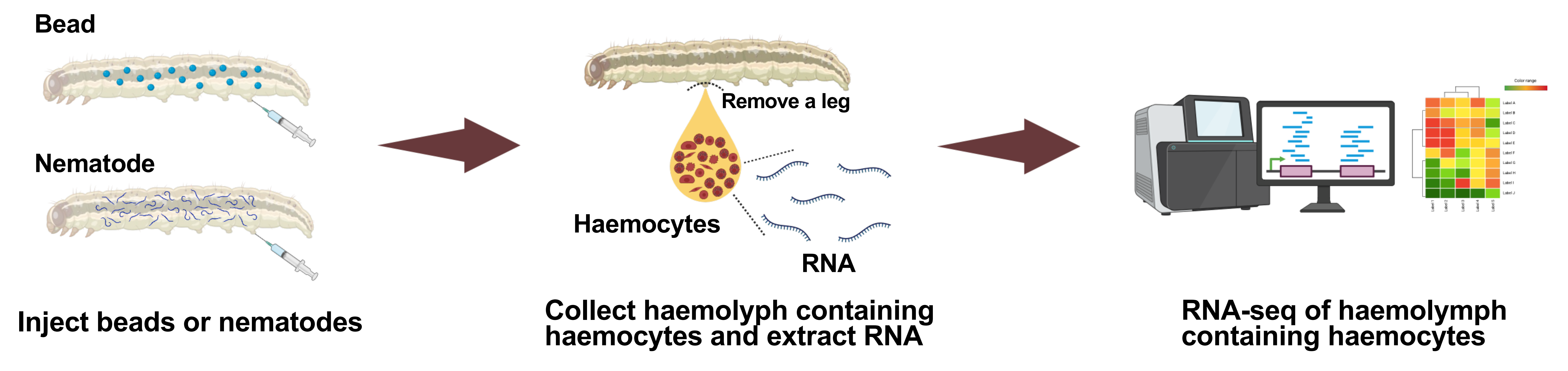
Bead
Nematode
Inject beads or nematodes
Remove a leg
Haemocytes
RNA
Collect haemolyph containing haemocytes and extract RNA
RNA-seq of haemolymph containing haemocytes
